# Impuesto especial a alimentos y bebidas y su impacto en la inflación en México: dinámica, persistencia y cambio de régimen

**DOI:** 10.26633/RPSP.2019.88

**Published:** 2019-11-15

**Authors:** Alfonso Mendoza-Velázquez, Dillan Aguirre Sedeño

**Affiliations:** 1 Centro de Investigación e Inteligencia Económica (CIIE) Universidad ­Popular Autónoma del Estado de Puebla (UPAEP) Puebla México Centro de Investigación e Inteligencia Económica (CIIE), Universidad ­Popular Autónoma del Estado de Puebla (UPAEP), Puebla, México.

**Keywords:** Impuestos, bebidas gaseosas, alimentos, inflación económica, México, Taxes, carbonated beverages, food, inflation, economic, Mexico, Impostos, bebidas gaseificadas, alimentos, inflação, México

## Abstract

**Objetivo.:**

El objetivo de esta investigación es estudiar la fuerza de traslado, la transición y la persistencia del impuesto especial sobre productos y servicios (IEPS) a alimentos y bebidas de densidad energética alta, sobre la tasa de inflación anual de estos alimentos y sobre las bebidas gaseosas, en vigencia desde enero de 2014.

**Métodos.:**

Se calcularon las tasas de inflación anualizadas para cada alimento y bebida desde enero de 2010 hasta diciembre de 2016 a partir de datos mensuales del índice nacional de precios al consumidor (INPC). Se empleó un modelo de regímenes cambiantes para estimar el impacto del impuesto sobre la dinámica inflacionaria de las bebidas y los alimentos, así como su transición, persistencia y las posibles rupturas.

**Resultados.:**

La dinámica inflacionaria de los alimentos de contenido calórico alto y las bebidas gaseosas sujetas a impuesto tiene una varianza alta. El impuesto fue trasladado a la inflación de algunos productos de densidad calórica alta de manera gradual, antes de regresar a los niveles inflacionarios previos al impuesto. La continuidad del impuesto no afecta la dinámica inflacionaria de los alimentos a partir de 2015.

**Conclusiones.:**

El impuesto debe ir acompañado de medidas que faciliten su persistencia y traslado a la dinámica inflacionaria de los productos con impuesto. La recaudación de estos impuestos debe fomentar y facilitar el consumo de alternativas saludables, así como acompañarlo de medidas educativas que modifiquen los hábitos de consumo de manera sostenida en el mediano plazo y el largo plazo.

## Abstract (en)

**Objective.:**

The objective of this research is to study the strength of transfer, the transitions, and the persistence of the special excise tax on products and services (known by its Spanish acronym, IEPS) applicable since January 2014 to high-energy foods and soft drinks, and the effect of the tax on the annual inflation rates for these foods and beverages.

**Methods.:**

Annual inflation rates for each food and beverage were calculated from January 2010 to December 2016, based on monthly data from the national consumer price index. A model of variable regimes was used to estimate the impact of the tax on the inflationary dynamics of the foods and beverages, as well as its transitions, persistence, and the possible ruptures.

**Results.:**

There was high variance in the inflationary dynamics of the high-calorie foods and soft drinks subject to the tax. The tax contributed gradually to price inflation for some high-calorie products, before returning to pre-tax inflation levels. The continuity of the tax did not affect the inflationary dynamics of the food after 2015.

**Conclusions.:**

The tax should be accompanied by measures that facilitate its persistence and transfer to the inflationary dynamics of the taxed products. The taxes collected should be used to promote and facilitate the consumption of healthy alternatives, and this should be accompanied by educational measures aimed at continuing to change consumption habits in the medium and long term.

## RESUMO

**Objetivo.:**

Estudar a força de transferência, transição e persistência do imposto especial sobre produtos e serviços (IEPS) para alimentos e bebidas de alta densidade calórica na taxa anual de inflação destes alimentos e refrigerantes, em vigor desde janeiro de 2014.

**Métodos.:**

As taxas anuais de inflação foram calculadas para cada alimento e bebida para o período de janeiro de 2010 a dezembro de 2016 com base em dados mensais do índice nacional de preços ao consumidor (INPC). Um modelo de mudança de regime foi usado para estimar a repercussão do imposto na dinâmica inflacionária de alimentos e bebidas bem como a transição, persistência e possíveis suspensões do imposto.

**Resultados.:**

A dinâmica inflacionária dos alimentos de alta densidade calórica e de refrigerantes sobre os quais incide o imposto apresenta grande variação. O imposto foi gradualmente transferido à dinâmica inflacionária de alguns produtos de alta densidade calórica e, posteriormente, houve um retorno aos níveis inflacionários anteriores à tributação. A continuidade do imposto não tem influenciado a dinâmica inflacionária dos alimentos desde 2015.

**Conclusões.:**

O imposto deve vir acompanhado de medidas para facilitar sua persistência e transferência à dinâmica inflacionária dos produtos tributados. A arrecadação destes impostos deve estimular e facilitar o consumo de alternativas de alimentos saudáveis, bem como vir acompanhado de medidas educativas para mudar os hábitos de consumo de forma sustentada a médio e longo prazo.

De acuerdo con datos de la Organización para la Cooperación y el Desarrollo Económicos (OCDE), México ocupa el primer lugar entre los países de la OCDE en prevalencia de obesidad entre adultos mayores (33,3%) y el cuarto en prevalencia de sobrepeso y obesidad tanto en niñas (35,1%) como en niños (34,9%), respectivamente, además de ser el país con mayor prevalencia de diabetes (1, 2). Estas condiciones representan un problema grave en materia de salud pública en México que se trasladarán en presiones fuertes para las finanzas públicas del país y para las familias en el mediano plazo y el largo plazo (3).

Una de las posibles causas de la prevalencia de sobrepeso y obesidad en niños y adultos se atribuye al consumo elevado de bebidas gaseosas (4) y de alimentos con contenido alto de grasas (5). Algunos enfoques de salud pública proponen la aplicación de impuestos especiales sobre el consumo como herramienta de política pública para combatir estos problemas (6). En el año 2014, el gobierno mexicano comenzó a gravar alimentos de contenido calórico alto y bebidas gaseosas con el impuesto especial sobre productos y servicios (IEPS).

La reforma hacendaria de ese año estableció un impuesto fijo de MXN 1 por litro a las bebidas saborizadas con endulzantes calóricos, así como un impuesto de 8% a los alimentos con contenido calórico igual o mayor a 275 kilocalorías por cada 100 gramos (7). Los productos gravados pertenecen a las siguientes categorías: botanas, productos de confitería, chocolates y productos derivados del cacao, flanes y pudines, dulces de frutas, cremas de cacahuate y avellanas, dulces de leche, dulces y confitería, alimentos preparados a base de cereales, helados, nieves y paletas de hielo, bebidas saborizantes y energizantes. Los productos no gravados considerados para este estudio por su contenido calórico alto son el azúcar, la manteca, el chorizo, el tocino, la manteca vegetal y las pizzas.

Este impuesto especial también tiene objetivos extrafiscales, puesto que buscan incidir en el consumo de la población (6) para disminuir problemas de salud tales como sobrepeso, obesidad y diabetes (8), al tiempo que se incrementan los ingresos fiscales (7). Para el caso mexicano en 2014, estos ingresos significaron un aumento de 51,7% con respecto al año anterior (9).

México no es el primer país en utilizar este tipo de impuestos. Países como Australia en el 2000, Fiyi en 2006, Naurú en 2007, Finlandia y Hungría en 2011, Francia en 2012 (10) y Barbados en 2017 (11) adoptaron impuestos especiales sobre el consumo de bebidas gaseosas. También lo implementaron en Estados Unidos (12), Bélgica, Dinamarca, Irlanda, Reino Unido y Tailandia, mientras que en América Latina lo adoptaron Brasil y Chile (13).

La teoría microeconómica muestra que los precios, más que los impuestos, tienen efectos sobre las decisiones de consumo de los consumidores (14), por lo que la efectividad del impuesto para reducir el consumo de los productos gravados dependerá de la magnitud y la persistencia del trasladado a los precios de mercado.

Haciendo uso de datos mensuales y de un modelo de datos panel con efectos fijos que incluye efectos estacionales, se ha encontrado que el impuesto se trasladó a los precios de las bebidas gaseosas de manera proporcional en las áreas urbanas de México (15), mientras que para productos con contenido calórico alto, el impacto del impuesto sobre el nivel de precios fue menos que proporcional. Haciendo uso de un método de control sintético con series de tiempo y de un análisis de intervención con datos mensuales por ciudad, Grogger muestra que en México el traslado del impuesto al precio de los refrescos fue mayor (16). En contraste, mediante el uso de un modelo de datos panel con efectos fijos semanales y control por estacionalidad, Aguilar et al. (9) monitorizaron el precio semanal de los productos gravados, y confirman que el traslado del impuesto a los precios de bebidas gaseosas fue menor, mientras que el traslado del impuesto a los precios de los productos con alto contenido calórico fue menor. La diferencia de traslado, es decir, cuánto del impuesto se refleja en la inflación o los precios, entre los estudios puede deberse al enfoque metodológico empleado, la frecuencia de los datos (semanal versus mensual) y la posible distinción por zonas urbanas.En Francia, se ha encontrado que el impuesto se traslada en forma proporcional al precio de los refrescos, mientras que para otro tipo de bebidas gaseosas el traslado del impuesto fue menor (10). Bergman y Lynggärd (8) analizaron un impuesto de este tipo sobre bebidas alcohólicas y no alcohólicas en Dinamarca y concluyeron que el impuesto se traslada a precios más que proporcionalmente. Por último, se ha reportado que el traslado del impuesto aplicado en Berkeley, California a precios fue menor (12).

Si bien hay evidencia creciente de que el impuesto se ha trasladado a los precios, aún falta determinar su impacto sobre la dinámica inflacionaria y si este es transitorio o persiste en el tiempo. Tampoco hay evidencia sobre si el traspaso del impuesto a los precios en México coincidió con la fecha de aplicación del impuesto, o si el traslado fue anticipado por el mercado, como ha sucedido en otros países (11). Existen pocos estudios que examinen si el impuesto genera quiebres o cambios de regímenes inflacionarios, así como la duración del impacto. Este estudio se refiere a la persistencia como la probabilidad de que la inflación económica permanezca en un régimen inflacionario alto o en uno bajo.

El objetivo de este estudio es analizar la fuerza del traslado, la transición y la persistencia del IEPS sobre la tasa de inflación anual de los productos de densidad energética alta y sobre las bebidas gaseosas, y evaluar la probabilidad de que el IEPS influya de manera temporal en la dinámica inflacionaria.

## MATERIALES Y MÉTODOS

### Materiales

Este es un estudio observacional que analiza el comportamiento longitudinal retrospectivo del crecimiento anual de los precios mensuales de cada uno de los grupos de productos gravados con el nuevo IEPS, desde su implementación en enero 2014 hasta diciembre de 2016. La inflación anualizada de cada alimento y bebida se calcula a partir del índice nacional de precios al consumidor (INPC) por objeto del gasto, reportado por el Instituto Nacional de Estadística y Geografía (INEGI) (17) con la siguiente fórmula:

$$\pi_{t}=(p_{t}/p_{t-12})-1$$

Donde:

*p_t_* representa el INPC de un alimento en un mes *t* específico

*p_t-12_* representa el INPC del mismo alimento 12 meses antes.

### Modelo de cambio de régimen inflacionario

El orden autorregresivo del modelo de Markov se elige con base en los criterios de información de Akaike (AIC) y Schwarz (SBIC). El modelaje incluye promedios condicionales para dos estados no observables y las varianzas.

El modelo de regresión de Markov permite analizar el comportamiento de las series de inflación anualizada en dos regímenes (“inflación baja” *s = 1* o “inflación alta” *s = 2*); el tiempo de transición de un estado a otro y la persistencia o duración de cada régimen inflacionario (18). El modelo de inflación anualizada (*π_t_*) de alimentos con densidad calórica alta es el siguiente:

$$\pi_{t}=\mu_{s_{t}}+\varepsilon_{s}$$

Donde:

*μs* es el promedio condicional de la inflación en cada uno de los estados latentes *s*, desconocidos a priori

*εs* es el error que se distribuye de forma idéntica e independiente como una normal con media 0 y varianza $\sigma_{s}^{2}.$

En caso de que la variación de la inflación anualizada no sea estable en el tiempo, también se estima la varianza para cada régimen *s*.

El modelo maximiza la siguiente función de log-­verosimilitud (19):

$$L(\theta )=\sum_{t=1}^{T}\ln f(\pi_{t}|\pi_{t-1},\;\theta )$$

Donde:

$\theta =\left[p_{ij},\;\mu_{s},\;\sigma_{s}^{2}\right]$ es el vector de parámetros a estimar

*π_t_* es la tasa de inflación anualizada en el tiempo *t*.

El modelo supone dos estados latentes *s* que determinan el valor de la inflación promedio a través de la siguiente matriz de probabilidades de transición condicionales:

$$P=\left[\begin{array}{cc} p_{11} & p_{12}\\ p_{21} & p_{22} \end{array}\right]$$

la cual se obtiene mediante una cadena de Markov (19). El valor individual *p_ij_* representa la probabilidad de transición del estado *i* al estado *j*. Por ejemplo, *p_12_* arroja la probabilidad de que el estado *1* sea seguido por el estado *2*. La persistencia en cada régimen puede calcularse como 1/(1-*p_ij_*), lo que arroja la duración promedio en meses de seguir en cada régimen.

## RESULTADOS

### Estadística descriptiva de la inflación anualizada

En el cuadro 1 se presenta estadística descriptiva de la inflación anualizada de los alimentos y bebidas. La inflación anualizada de los alimentos con impuesto presenta un rango que va desde 2,64% en las bebidas concentradas, hasta 36,01% en el pan dulce, producto que presenta también la mayor variación temporal (desviación estándar: 4,63%), seguido de la variación de los refrescos con 4,42%. Entre los productos sin impuesto, destaca el azúcar con la mayor variación con una desviación estándar de 19,52%, pero una inflación promedio de 1,34%.

### Estimaciones del modelo

En los cuadros 2 y 3 se presentan las estimaciones de los efectos de traslado transición y persistencia de la inflación, antes y después de la introducción del IEPS sobre los alimentos y bebidas en este estudio. Todos los promedios condicionales de la inflación anualizada estimados (*μ_1_* y *μ_2_*) son estadísticamente significativos, salvo para el estado de inflación baja (*s = 1)* de las bebidas concentradas, la leche condensada y la gelatina. En el estado *s = 1* (primera línea de los cuadros 2 y 3), el azúcar presenta períodos deflacionarios fuertes y significativos (-20,09%), en contraste con el resto de los promedios de inflación anualizada (*μ_1_*) que muestran inflaciones bajas, pero positivas, con un rango que va de -2,46% (manteca vegetal) a 5,43% (tocino). En el estado de inflación alta (*s = 2),* se registran rangos de inflación anual que van desde 3,86% (gelatina) hasta 13,15% (panecillos), 14,46% (azúcar) y 14,94% (refrescos).

### Persistencia y duración de los regímenes inflacionarios

Los paneles de probabilidad de transición de los cuadros 2 y 3 muestran que la probabilidad de permanecer en un estado inicial de inflación baja es cercana a 1 en todos los alimentos, lo que implica que la probabilidad de que un mes de inflación baja sea seguido por otro mes de inflación baja es muy alta (en promedio, 96,00%): el régimen de inflación baja tiene una persistencia promedio de 1/(1-*p_11_*) = 25 meses (ver panel de duración promedio en los cuadros 2 y 3). La probabilidad de que un régimen de inflación baja (estado *s = 1*) sea seguido por un régimen de inflación alta (estado *s = 2*) alcanza, por tanto, *p_12_* = 4,00%, en promedio. De manera similar, la probabilidad de que un mes de inflación alta sea seguido por otro mes de inflación alta resulta *p_22_* = 93,53%, con una persistencia de 1/(1-*p_22_*) = 15,46 meses, en promedio. De la misma forma, la probabilidad de pasar al régimen de inflación baja después de haber permanecido en un régimen de inflación alta alcanza *p_21_* = 6,47% en promedio.

**CUADRO 1.** Estadística descriptiva de la inflación anualizada (enero 2010-diciembre 2016)

Producto	Promedioa	DE	Mínimo	Máximo	Sesgo	Curtosis
Azúcar	1,34%	19,52%	-36,17%	33,75%	-0,40	2,07
Bebidas concentradas (T)^c^	2,64%	3,50%	-3,12%	11,19%	0,36	2,39
Cajetas y mermeladas (T)	4,47%	2,21%	0,19%	9,11%	0,46	2,36
Chorizo	5,70%	2,74%	1,52%	12,29%	0,60	2,34
Dulces (T)	4,87%	3,88%	-0,83%	13,52%	0,60	2,25
Frituras (T)	5,11%	3,10%	1,09%	11,17%	0,05	2,30
Galletas (T)	4,42%	2,10%	0,82%	9,67%	0,54	2,78
Gelatinas (T)	2,71%	2,49%	1,84%	9,21%	0,31	2,55
Helados (T)	4,21%	2,53%	0,06%	10,84%	0,73	3,14
Leche condensada (T)	3,05%	3,07%	-5,02%	9,06%	-0,21	2,86
Manteca	3,84%	5,20%	-0,64%	14,78%	0,25	2,46
Manteca vegetal	2,77%	6,05%	-4,92%	15,87%	0,66	2,41
Mayonesa	3,91%	3,11%	1,87%	10,28%	0,22	2,41
Pan dulce (T)	36,01%	4,63%	-3,08%	13,10%	-0,55	2,08
Panecillos (T)	6,32%	3,82%	-0,10%	15,32%	0,57	2,82
Pasteles (T)	5,37%	2,96%	-0,37%	10,53%	-0,22	1,98
Pizza	3,08%	1,69%	0,18%	6,99%	0,10	2,23
Refrescos (T)	5,21%	4,42%	1,97%	15,94%	1,74	4,15
Tocino	7,69%	3,38%	1,04%	16,31%	0,23	2,69

***Fuente:*** elaboración propia con datos de la INPC mensual por objeto del gasto reportado por el INEGI (17).

^a^ El cálculo del promedio utiliza 72 observaciones.

DE, desviación estándar; (T), indica que el producto está sujeto a impuesto.

**CUADRO 2.** Estimaciones del modelo de cambio de régimen inflacionario (enero 2010-diciembre 2016) para azúcar, refrescos, pan dulce, panecillos, pasteles, galletas, manteca, chorizo y tocino

Resultados	Azúcar	Refrescos (T)	Bebidas (T)	Pan dulce (T)	Panecillos (T)	Pasteles (T)	Galletas (T)	Manteca	Chorizo	Tocino
Régimen (promedios condicionales μ1 y μ2)										
Estado *s = 1*	-0,2009***^d^	0,0326***	0,0023	0,0107*	0,0495***	0,0322***	0,0368***	0,0082**	0,0399***	0,0543***
[Promedio de baja inflación]	(0,0284)^e^	(0,0008)	(0,0105)	(0,0059)	(0,0032)	(0,0054)	(0,0017)	(0,0048)	(0,0022)	(0,0056)
Estado *s = 2*	0,1446***	0,1494***	0,0596***	0,0931***	0,1315***	0,0822***	0,0816***	0,1003***	0,0904***	0,1061***
[Promedio de alta inflación]	(0,0176)	(0,0016)	(0,0139)	(0,0029)	(0,0042)	(0,0043)	(0,0018)	(0,0067)	(0,0041)	(0,0068)
Varianzas										
Sigma^a^	0,0973	0,0059	0,0201	0,0274	0,0224	0,0189	0,0125	0,0282	0,0130	0,0217
	(0,0073)	(0,0004)	(0,0019)	(0,0025)	(0,0019)	(0,0025)	(0,0009)	(0,0028)	(0,0013)	(0,0018)
Sigma(2)^b^	-	-	-	0,0174	-	0,0108	-	-	-	-
				(0,0016)		(0,0028)				
Probabilidades de transición										
P_1_,_1_^c^	0,9471	0,9853	0,9552	0,9495	0,9852	0,9583	0,9852	0,9627	0,9629	0,9314
P_1_,_2_	0,0529	0,0147	0,0448	0,0505	0,0148	0,0417	0,0148	0,0373	0,0371	0,0686
	(0,0352)	(0,0153)	(0,0323)	(0,0239)	(0,0111)	(0,0240)	(0,0154)	(0,0201)	(0,0211)	(0,0333)
P_2_,_1_	0,0361	0,0936	0,0803	0,0373	0,0937	0,0756	0,0939	0,0957	0,0928	0,0848
P_2_,_2_	0,9639	0,9064	0,9197	0,9627	0,9063	0,9244	0,9061	0,9043	0,9072	0,9152
	(0,0168)	(0,0744)	(0,0443)	(0,0267)	(0,0561)	(0,0375)	(0,0738)	(0,0393)	(0,0383)	(0,0330)
Duración (persistencia) promedio del régimen en meses										
Inflación baja	18,9117	68,1006	22,3340	19,8025	67,7584	23,9759	67,6589	28,8249	26,9901	14,5876
	(12,6021)	(70,9189)	(16,1254)	(9,3897)	(50,8443)	(13,8141)	(70,3944)	(14,4792)	(15,3546)	(7,0823)
Inflación alta	27,7008	10,6862	12,4592	26,8383	10,6727	13,2327	10,6460	10,4465	10,7772	11,7904
	(12,8602)	(8,4924)	(6,8832)	(19,2271)	(6,3862)	(6,5650)	(8,3664)	(4,2944)	(4,4439)	(4,5943)
Criterios de decisión										
Log verosimilitud	55,7190	258,6035	167,4424	164,5797	162,9166	188,2442	204,9204	142,3791	197,4731	161,4526
AIC	-101,438	-507,2069	-324,8847	-317,1594	-315,8333	-364,4885	-399,8407	-274,7582	-384,9463	-312,9052
BIC	-90,0547	-495,8236	-313,5014	-303,4994	-304,4499	-350,8285	-388,4574	-263,3749	-373,5629	-301,5219

***Fuente:*** Elaboración y estimaciones propias en base a datos del INEGI (17).

^a^Desviación estándar entre estados.

^b^En los casos donde aplique, cada sigma hace referencia a la desviación estándar en el estado de baja inflación y en el estado de alta inflación, respectivamente.

^c^Probabilidad incondicional de comenzar en el estado i y terminar en el estado j.

^d^El nivel de significancia de la prueba t para probar H0: bi = 0 se indica con *P < 0,1, **P < 0,05 y ***P < 0,01.

^e^Errores estándares robustos entre paréntesis.

(T), productos con impuesto; AIC, criterio de información de Akaike (por sus siglas en inglés); BIC, criterio de información de Bayes (por sus siglas en inglés).

**CUADRO 3.** Estimaciones del modelo de cambio de régimen inflacionario, enero 2010-diciembre 2016 para manteca vegetal, leche condensada, helados, mayonesa, frituras, dulces, cajetas y mermeladas, gelatina y pizza

Resultados	Manteca vegetal	Leche condensada (T)	Helados (T)	Mayonesa	Frituras (T)	Dulces (T)	Cajetas y mermeladas (T)	Gelatina (T)	Pizza
Régimen (promedios condicionales μ1 y μ2)									
Estado *s = 1*	-0,0246***	0,0019	0,0312***	0,0180***	0,0319***	0,0216***	0,0332***	-0,0011	0,0196***
[Promedio de baja inflación]	(0,0052)	(0,0048)	(0,0023)	(0,0036)	(0,0049)	(0,0024)	(0,0024)	(0,0036)	(0,0017)
Estado *s = 2*	0,0734***	0,0504***	0,0799***	0,0682***	0,0834***	0,0896***	0,0738***	0,0386***	0,0473***
[Promedio de alta inflación]	(0,0103)	(0,0039)	(0,0049)	(0,0043)	(0,0067)	(0,0051)	(0,0044)	(0,0030)	(0,0016)
Varianzas									
Sigma^a^	0,0170	0,0191	0,0148	0,0184	0,0181	0,0152	0,0120	0,0010	0,0097
	(0,0029)	(0,0017)	(0,0013)	(0,0013)	(0,0013)	(0,0015)	(0,0011)	(0,0023)	(0,0008)
Sigma(2)^b^	0,0452	-	-	-	-	0,0239	-	0,0192	-
	(0,0043)					(0,0025)		(0,0019)	
Probabilidades de transición									
P_1_,_1_^c^	0,9610	0,9562	0,9686	0,9612	0,9599	0,9870	0,9465	0,8899	0,9868
P_1_,_2_	0,0390	0,0438	0,0314	0,0388	0,0401	0,0130	0,0535	0,1101	0,0132
	(0,0230)	(0,0210)	(0,0158)	(0,0210)	(0,0265)	(0,0079)	(0,0280)	(0,0460)	(0,0172)
P_2_,_1_	0,0184	0,0184	0,0842	0,0497	0,0867	0,0159	0,1199	0,0369	0,0161
P_2_,_2_	0,9816	0,9816	0,9158	0,9503	0,9133	0,9841	0,8801	0,9631	0,9839
	(0,0126)	(0,0153)	(0,0482)	(0,0243)	(0,0443)	(0,0204)	(0,0623)	(0,0190)	(0,0109)
Duración (persistencia) promedio del régimen en meses									
Baja inflación	25,64	22,82	31,82	25,77	24,93	77,24	18,68	9,08	75,68
	(15,05)	(10,94)	(16,04)	(13,98)	(16,494)	(47,08)	(9,75)	(3,79)	(98,59)
Alta inflación	54,31	54,35	11,88	20,10	11,54	62,92	8,34	27,06	61,95
	(37,06)	(45,34)	(6,81)	(9,83)	(5,89)	(80,70)	(4,33)	(13,94)	(41,89)
Criterios de decisión									
Log verosimilitud	146,80	176,80	190,50	175,90	174,62	181,30	201,28	184,32	226,05
AIC^g^	-281,60	-343,59	-370,99	-341,80	-339,24	-350,60	-392,55	-356,64	-442,09
BIC^h^	-267,94	-332,21	-359,62	-330,42	-327,85	-336,940	-381,17	-342,98	-430,71

***Fuente:*** Elaboración y estimaciones propias en base a datos del INEGI (17).

^a^Desviación estándar entre estados.

^b^En los casos donde aplique, cada sigma hace referencia a la desviación estándar en el estado de baja inflación y en el estado de alta inflación, respectivamente.

^c^Probabilidad incondicional de comenzar en el estado i y terminar en el estado j.

^d^El nivel de significancia de la prueba t para probar H0: bi = 0 se indica con *P < 0,1, **P < 0,05 y ***P < 0,01.

^e^Errores estándares robustos entre paréntesis.

(T), productos con impuesto; AIC, criterio de información de Akaike (por sus siglas en inglés); BIC, criterio de información de Bayes (por sus siglas en inglés).

**FIGURA 1.**
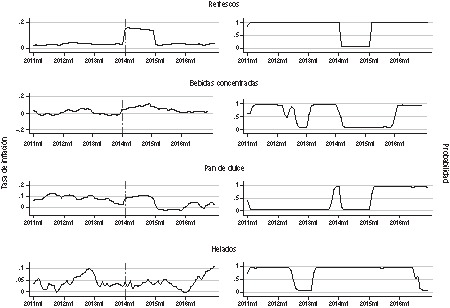
Inflación anualizada y probabilidad del régimen de baja inflación por producto (alimentos y bebidas selectos)

***Fuente:*** Elaboración y estimaciones propias con datos de precios mensuales del INEGI (17).

Nota: m1 en el eje horizontal se refiere al mes de enero de cada año.

Los resultados en los cuadros 2 y 3 también muestran cinco casos con cambios de varianza estadísticamente significativos: pan dulce, pasteles, manteca vegetal, dulces y gelatina.

En las figuras 1 y 2 se muestra el comportamiento de la inflación anualizada de productos seleccionados en el período de estudio (primera columna), así como la probabilidad no condicional de permanecer en el estado de inflación baja (segunda columna). La inflación anualizada de los refrescos, por ejemplo, responde de inmediato a la aplicación del impuesto, lo que eleva la inflación de porcentajes menores a 5% (estado *s = 1*) a valores inflacionarios cercanos a 15% (estado *s = 2*).

## DISCUSIÓN

En este estudio se estima la fuerza de traslado, la transición y la persistencia de la inflación anualizada después de la implementación del IEPS mediante un modelo de Markov de cambio de regímenes a partir de índices de precios disponibles para el público. El estudio encuentra que la dinámica inflacionaria de los alimentos de contenido calórico alto y bebidas gaseosas sujetas a impuesto tiene una varianza alta; que la inflación de varios alimentos aumentó incluso antes de la introducción del impuesto y que el régimen de inflación baja es muy persistente, ya que muestra una duración promedio mayor que el régimen de inflación alta.

La inflación anualizada de los productos sin impuesto mostró una dinámica sin rupturas alrededor de la fecha de aprobación del impuesto (manteca vegetal y mayonesa), lo que sugiere que la dinámica inflacionaria de estos productos no se relaciona en forma directa con el impuesto. La mayoría de los productos gravados en la muestra trasladaron el impuesto a la dinámica inflacionaria de los precios meses antes de la entrada en vigor del impuesto nuevo a bebidas y alimentos de contenido calórico alto (11). La evolución de la inflación anualizada (panel izquierdo) y la transición de cambio de régimen (probabilidades en panel derecho) de las figuras 1 y 2 confirman que, para algunos artículos, el traslado del impuesto a la inflación ocurrió antes, aproximadamente en noviembre de 2013, fecha de aprobación de la Reforma Hacendaria al IEPS, aunque el consenso social sobre la necesidad del impuesto inició mucho antes (20). Obsérvense los puntos de inflexión en las series de inflación de las bebidas concentradas, la pizza, las cajetas y las mermeladas. El IEPS se trasladó de manera notoria a la dinámica inflacionaria de los refrescos, pan dulce, panecillos, pasteles, galletas, frituras, cajetas y mermeladas. La dinámica inflacionaria de estos productos se ubica históricamente en regímenes de inflación baja. La aprobación del IEPS en noviembre de 2013 y su aplicación en enero de 2014 forzó la ruptura del régimen de inflación baja de estos productos y detonó el cambio a un régimen de inflación alta. La probabilidad de permanecer en niveles de inflación baja es más alta para productos gravados como los refrescos (98,53%), los panecillos (98,52%), las galletas (98,52%) y los dulces (98,70%), al tiempo que estos productos muestran las probabilidades más bajas de mantenerse en regímenes de inflación alta. El impuesto se trasladó a la dinámica inflacionaria de manera gradual, mes a mes, como lo muestra la reacción de la inflación anualizada de estos productos, para al final regresar al estado habitual de inflación baja. El período de inflación alta de los refrescos también es transitorio y, a partir de enero de 2015, regresa a los niveles preimpuesto, con inflaciones menores de 5%. El comportamiento de las series de inflación anualizada, de las estimaciones de las probabilidades de transición y de la persistencia, no se vieron afectadas con la continuación del impuesto año con año desde 2015. No se registran cambios de régimen significativos que puedan atribuirse a la aplicación del IEPS en fechas posteriores a la primera vez que se introdujo el impuesto en enero de 2014.

**FIGURA 2.**
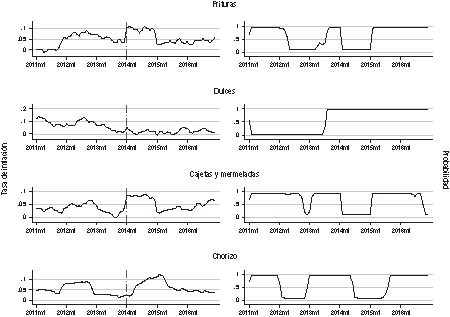
Inflación anualizada y probabilidad del régimen de baja inflación por producto (alimentos y bebidas selectos)

***Fuente:*** Elaboración y estimaciones propias en base a datos de precios mensuales del INEGI (16).

Nota: m1 en el eje horizontal se refiere al mes de enero de cada año.

Estos hallazgos sugieren que la efectividad del impuesto para afectar la inflación de los refrescos y de productos de alto contenido calórico es transitoria; es decir, la inflación de los productos gravados retorna a niveles preimpuesto en el corto plazo. El comportamiento habitual de inflación baja en los productos examinados en este estudio puede ser resultado de la adopción del esquema de objetivos de inflación económica de 3%, implementado por el Banco de México, que arrastra a estos alimentos hacia la tendencia seguida para controlar el crecimiento del nivel general de precios.

Los resultados de este estudio complementan los reportes de diversos estudios sobre el impacto de los impuestos sobre el nivel de precios y la inflación de los alimentos de alto contenido calórico y de bebidas gaseosas (8, 14, 21). También concuerdan con una investigación reciente para Chile que sugiere que los impuestos específicos, como el de las bebidas gaseosas, puede diluirse con el tiempo. Por esta razón se sugiere que los impuestos específicos se indexen a la inflación (21). El impuesto *ad valorem,* aplicado a los alimentos con contenido calórico alto, debería ajustarse en forma automática con la inflación, pero este estudio encuentra evidencia mixta, por ejemplo, la inflación anualizada de los helados y los dulces no reaccionan al impuesto, mientras que la inflación del pan dulce, las frituras y las cajetas reaccionan de forma inmediata.

La inflación de los alimentos es uno de los componentes más volátiles de la inflación general de precios y quizá el más regresivo (21), ya que afecta de manera más directa a los estratos sociales de menores ingresos. Por esta razón se ha sugerido que el ingreso fiscal generado se reintegre a los consumidores en forma de agua potable en escuelas públicas o programas educativos para mejorar los hábitos de consumo (20).

El comportamiento de precios y la transmisión de la inflación de los alimentos domésticos tienen un origen multifactorial: estructura de competencia, precios internacionales y política monetaria, entre otros (22, 23). El esquema de objetivos de inflación adoptado por diversos países, muchos de los cuales han también aplicado impuestos a los alimentos de contenido calórico alto y bebidas gaseosas, busca frenar el crecimiento de los precios al establecer metas de inflación subyacente anuales. Una investigación reciente ha mostrado que la inflación de los alimentos y la inflación subyacente muestran efectos de propagación y retroalimentación importantes en países emergentes, donde la proporción de los alimentos en las canastas de consumos es muy alta (24). De esta forma, los resultados sobre la transición y persistencia del cambio de un régimen inflacionario a otro reportados en este estudio son relevantes para otros países emergentes con esquemas de objetivos de inflación que buscan acabar con el sobrepeso y la obesidad a través de impuestos como los aplicados en México.

La probabilidad alta de mantenerse en regímenes de inflación baja implica que el impacto del impuesto es muy transitorio. Este resultado sugiere que la capacidad de respuesta de la industria para contener el traslado de los precios a sus productos es eficiente y, tal vez, que el cambio hacia conductas sanas de los consumidores también puede ser transitorio. La respuesta de las empresas al impuesto debe estudiarse con mayor precisión, así como sus mecanismos de ajuste y absorción de precios y los impactos sobre la inflación de los alimentos de contenido calórico alto y de bebidas gaseosas. De manera inicial, la estructura oligopólica del mercado puede explicar el traslado diferencial del impuesto a los precios (6).

Los índices empleados en este estudio limitan la distinción del impacto del impuesto sobre el crecimiento de los precios de los alimentos por tipo de producto, tamaños, presentaciones, calidad o por tipo de tienda (21). Sin embargo, los hallazgos en este estudio distinguen características no estudiadas antes en la literatura, como la probabilidad de cambio de régimen inflacionario alrededor del impuesto, así como su persistencia o duración en cada régimen inflacionario. Estudios futuros pueden considerar el rol de variables de estructura de mercado, la distancia y del manejo de la política monetaria.

La ausencia de datos de sobrepeso y obesidad por mes no permite evaluar el impacto del impuesto, de los precios y de la inflación de los alimentos y bebidas gravados. No se pueden establecer ligas causales entre la implementación de impuestos a bebidas gaseosas y alimentos de contenido calórico alto y variables de salud (21, 25). Los cambios inflacionarios sugieren cambios de comportamiento anticipados por parte de la industria alimenticia y las refresqueras que, sin embargo, tampoco han logrado medirse en este estudio.

## CONCLUSIONES

Esta es la primera investigación acerca del traslado, la transición y la persistencia del cambio de régimen inflacionario de las bebidas gaseosas y alimentos de contenido calórico alto después del impuesto implementado en México en enero de 2014. El estudio encuentra que el traslado de la inflación es solo transitorio y que la probabilidad de cambio a un régimen de inflación baja es muy alta. El trasladado del impuesto a la inflación de estos productos se distribuye de manera gradual y es poco persistente, antes de regresar a niveles inflacionarios preimpuesto. La continuación del impuesto no afecta la dinámica inflacionaria de los alimentos a partir de 2015 y debería ir acompañada de medidas que aseguren el traslado y la persistencia del impuesto a la dinámica de los precios de los productos específicos y en los puntos de venta. Las autoridades sanitarias que busquen implementar este tipo de impuestos deberían aprovechar el impacto sobre el precio y la inflación, sobre todo en los primeros meses de su aplicación, para fomentar y facilitar el consumo de alternativas saludables como el agua potable, así como acompañarlo de medidas educativas que modifiquen los hábitos de consumo de manera sostenida en el mediano y largo plazo. Con el fin de amortiguar efectos recesivos, los ingresos fiscales captados por los dos tipos de impuesto deben reintegrarse a los consumidores facilitando el acceso a fuentes de agua potable o programas educativos para mejorar los hábitos de consumo de los habitantes.

### Contribución de los autores.

AM concibió la idea del estudio original. DA planificó y recolectó los datos. AM y DA interpretaron los resultados, escribieron y revisaron el manuscrito. Todos los autores revisaron y aprobaron la versión final.

### Declaración.

Las opiniones expresadas en este manuscrito son responsabilidad de los autores y no reflejan necesariamente los de la *Revista Panamericana de Salud Pública* o la Organización Panamericana de la Salud.
